# Plastoquinone pool redox state and control of state transitions in *Chlamydomonas reinhardtii* in darkness and under illumination

**DOI:** 10.1007/s11120-022-00970-3

**Published:** 2022-10-25

**Authors:** Olli Virtanen, Esa Tyystjärvi

**Affiliations:** Department of Life Technologies/Molecular Plant Biology, University of Turku, 20014 Turku, Finland

**Keywords:** Anaerobicity, Chlamydomonas reinhardtii, Dark incubation, Monochromatic light, Plastoquinone pool, White light

## Abstract

Movement of LHCII between two photosystems has been assumed to be similarly controlled by the redox state of the plastoquinone pool (PQ-pool) in plants and green algae. Here we show that the redox state of the PQ-pool of *Chlamydomonas reinhardtii* can be determined with HPLC and use this method to compare the light state in *C. reinhardtii* with the PQ-pool redox state in a number of conditions. The PQ-pool was at least moderately reduced under illumination with all tested types of visible light and oxidation was achieved only with aerobic dark treatment or with far-red light. Although dark incubations and white light forms with spectral distribution favoring one photosystem affected the redox state of PQ-pool differently, they induced similar Stt7-dependent state transitions. Thus, under illumination the dynamics of the PQ-pool and its connection with light state appears more complicated in *C. reinhardtii* than in plants. We suggest this to stem from the larger number of LHC-units and from less different absorption profiles of the photosystems in *C. reinhardtii* than in plants. The data demonstrate that the two different control mechanisms required to fulfill the dual function of state transitions in *C. reinhardtii* in photoprotection and in balancing light utilization are activated via different means.

**Supplementary Information:**

The online version contains supplementary material available at 10.1007/s11120-022-00970-3.

## Introduction

In photosynthetic light reactions, plastoquinone (PQ), cytochrome *b*_*6*_*f* complex (cyt *b*_*6*_*f*) and plastocyanin carry electrons from Photosystem II (PSII) to Photosystem I (PSI). Oxidized and reduced forms of plastoquinone (PQ, PQH_2_) form the photochemically active thylakoid pool (PQ-pool), which in both plants (Lichtenthaler et al. 1981; Kruk and Karpinski 2006; Mattila et al. 2020) and cyanobacteria (Khorobrykh et al. 2020) makes up for only fraction of total PQ. In plants, the remaining PQ is stored in the inner chloroplast envelope and plastoglobuli (for review see van Wijk and Kessler 2018), where it stored as PQH_2_ (Piller et al. 2011).

Green algae have more light-harvesting capacity than plants. *Chlamydomonas reinhardtii* has nine genes coding for PSII-binding Major Light-Harvesting-Complexes (LHCBMs) (*Lhcbm*1-9) (Minagawa and Takahashi 2004), whereas *Arabidopsis thaliana* only has three (Jansson 1999). Albeit highly homologous, the different LHCBMs in *C. reinhardtii* have been reported to have specific functions (for review see Wobbe et al. 2016). The antenna of PSII in *C. reinhardtii* is larger than in plants, mostly due to replacement of monomeric CP24 with a trimeric LHCII in *C. reinhardtii* (Minagawa and Takahashi 2004; Sheng et al. 2021). In addition, *C. reinhardtii* has ten Lhca proteins per PSI core (Kubota-Kawai et al. 2019; Su et al. 2019) while *A. thaliana* has four (Croce et al. 2002). Furthermore, the PSI-LHCI complex of *C. reinhardtii* has much lower chlorophyll (Chl) *a/b* ratio (Drop et al. 2014a) than the PSI-LHCI of *A. thaliana* (Galka et al. 2012) and *Pisum sativum* (van Oort et al. 2008; Caspy and Nelson 2018).

To balance light utilization especially in low light, green algae and plants move parts of LHCII between PSII and PSI in the state transition mechanism associated with phosphorylation of LHCII (Mullineaux and Emlyn-Jones 2004; Tikkanen et al. 2006). The STN7-kinase responsible for LHCII-phosphorylation in plants is activated via binding of PQH_2_ to the Q_0_-site of cyt *b*_*6*_*f* (Vener et al. 1995, 1997; Zito et al. 1999; Bellafiore et al. 2005; Shapiguzov et al. 2016; Dumas et al. 2017), and the light state depends curvilinearly on the PQ-pool redox state in *A. thaliana* (Mattila et al. 2020). This enables the redox state of the PQ-pool to mediate state transitions in plants by sensing imbalance of electron transfer rates (Allen et al. 1981; Mattila et al. 2020).

A mechanism similar to STN7-dependent state transitions has been assumed to function in green algae, as their LHCII-phosphorylating enzyme, Stt7, is a close orthologue of STN7 (Fleischmann et al. 1999; Depége et al. 2003; Bellafiore et al. 2005; Lemeille et al. 2010) and is also activated by Q_0_-site occupancy (Finazzi et al. 2001; Depége et al. 2003). However, even though 80% of LHCII units are capable of energetically detaching from PSII in *C. reinhardtii* (Delosme et al. 1996), only a small fraction of LHCII units has been shown to attach to PSI (Takahashi et al. 2013; Nagy et al. 2014; Ünlü et al. 2014) and the majority of PSII-LHCII supercomplexes have been suggested to remain physically intact in State 2 (Minagawa and Tokutsu 2015).

State transition in green algae can be expected to be more complex than the phosphorylation-dependent movement of LHCII in plants for three reasons. Firstly, the Light-Harvesting-Complex-Stress-Related Proteins (LHCSRs) are involved in state transitions, in addition to modulating nonphotochemical quenching of PSII excitation energy (Bonente et al. 2011; Ferrante et al. 2012; Roach and Na 2017; Tian et al 2019), which has led to a suggestion that state transitions play a photoprotective role in green algae (Wobbe et al. 2016). Secondly, cyclic electron flow is activated in green algae in conditions promoting transition to State 2 (Finazzi et al. 2002; Steinbeck et al. 2018). Thirdly, state transitions are linked to nonphotochemical metabolism in *C. reinhardtii,* (Cardol et al. 2003), and in fact, State 2 is traditionally induced via the combination of darkness and anaerobicity, conditions that disable both mitochondrial respiration and chlororespiration (Rebeille and Gans 1988; Bulté et al. 1990). Under these conditions, the PQ-pool is effectively reduced by Type II NAD(P)H dehydrogenase (Nda2) (Jans et al. 2008), which enables transition to State 2. The contribution of Nda2 in PQ-pool reduction in the light is not clear but since it functions light-independently (Jans et al. 2008), it can be assumed to function also under illumination.

Only indirect methods have earlier been used to estimate the redox state of the PQ-pool in green algae. Here, we applied a method designed for the direct measurement of the redox state of the PQ-pool of plants (Kruk and Karpinski 2006) with the modifications that allowed its application to cyanobacteria (Khorobrykh et al. 2020). The size of the photochemically active PQ-pool was measured and an action spectrum of its redox state was determined using the same light wavelengths as earlier used for *A. thaliana* (Mattila et al. 2020). The relationship between the PQ-pool redox state and the light state was studied by inducing state transitions both by traditional dark treatments and by treatments with white PSII or PSI light, obtained by combining three wavelengths that respectively reduce or oxidize the PQ-pool. The effect of light intensity was inspected under illumination by the monochromatic components of the white PSII and PSI light.

## Materials and methods

### Algal strains and cultures

The majority of the experiments were done with a commonly used laboratory strain of *Chlamydomonas reinhardtii*, *cc124* (mt-)*.* This strain was used as a control for the state transition-deficient mutant *stt7-9* (mt-) (Cardol et al. 2009), originally generated from the arginine-deficient cell wall less strain of *C. reinhardtii* (Fleischmann et al. 1999). The *stt7-9* mutant was kindly provided to us by Roberta Croce. Cells were grown photoautotrophically in high salt (HS) medium (Sueoka 1960), at 27 °C, at continuous PPFD of 100 or 50 µmol m^−2^ s^−1^, as indicated, and in ambient air supplied with 1% CO_2_. Aliquots for the treatments were collected during mid-to-late exponential growth phase, determined via optical density at 730 nm (OD_730_). Biological replicates in the experiments refer to sample subset of cells taken from individual subcultures, grown from very low cell density in their individual inocula, originating form a common base population of cells. Technical replicates refer to repetitions of measurements conducted with same subpopulation of cells.

### Plastoquinone measurements

The redox state of the PQ-pool of *C. reinhardtii* was measured with a method designed for plants (Kruk and Karpinski 2006; Mattila et al. 2020) with modifications for cyanobacteria (Khorobrykh et al. 2020). Samples were prepared by filtrating approximately 10–15 × 10^6^ cells, determined spectrophotometrically from a known relationship between OD_730_ and cell density (calculated with a cell counting chamber), on a glass microfiber filter with pore size of 1.6 µm (VWR, USA, Cat. No. VWRI516-0862). All illumination treatments were done to cells on the filters. PQ was always rapidly extracted by grinding the filter in a mortar in dry ice-cold ethyl acetate under the respective treatment light or in the dark for dark treatments. The further preparation of the samples for HPLC was done as described by Khorobrykh et al. (2020). The redox state of total PQ was measured from two HPLC samples, one run without and one with addition of 5 mM of NaBH_4_.

For the estimation of the size of the photochemically active PQ-pool, the redox state of the extracted PQ was measured after fully oxidizing and after fully reducing light treatments. For maximal oxidation, cells were treated with far-red light (> 700 nm) with the photon flux density (PFD) of 50 µmol m^−2^ s^−1^ for 10 min (see Fig. S1 for the spectra). PFD was calculated from measurement with an STS-VIS spectrometer (Ocean Insight, Ostfildern, Germany, D-73760). For maximal reduction, cells were illuminated for 30 s with strong white light with photosynthetic photon flux density (PPFD) 2000 µmol m^−2^ s^−1^.

To test alternative methods for PQ-pool reduction and oxidation, treatments were repeated in the presence of 5 µM 2,5-dibromo-6-isopropyl-3-methyl-1,4-benzoquinone (DBMIB) and 20 µM 3-(3,4-dichlorophenyl)-1,1-dimethylurea (DCMU), respectively. These artificial quinones were added 1 min prior to the illumination with either far-red (DCMU) or high light (DBMIB) as described above. After establishing the PQH_2_/(PQH_2_ + PQ) ratio at full reduction and oxidation of the photochemically active PQ-pool, the redox state of the PQ-pool of an any sample could then be calculated from Eq. (1) (Kruk and Karpinski 2006) as

1 $$\% of PQ-pool reduced = 100 \times \left( {F_{SAMPLE} - F_{OXIDIZED} } \right)/\left( {F_{REDUCED} - F_{OXIDIZED} } \right),$$

where *F*_*SAMPLE*_ represents the ratio PQH_2_/(PQ + PQH_2_) in an unknown sample, obtained by measuring an aliquot of the same sample with and without addition of NaBH_4_ that reduces all PQ in the sample. *F*_*OXIDIZED*_ and *F*_*REDUCED*_ refer to the same ratio in reference data obtained after full oxidation of the PQ-pool with far-red light treatment and after full reduction of the PQ-pool with a short treatment with high light, respectively.

PQ-pool redox state was examined from the growth conditions by illuminating cells on a filter with the respective growth PPFD, 100 or 50 µmol m^−2^ s^−1^, as indicated, for 5 min.

Custom-built white light sources favoring PSII or PSI were used. These types of white light were obtained by combining equal PFD of either 430, 520 and 690 nm narrow-band light (PSI light) or 470, 560 and 660 nm light (PSII light) (See Fig. S2a for spectra). LEDs equipped with 10 nm half-width at half maximum optical filters transmitting 430, 470, 520, 560, 660 or 690 nm light (Andover Corporation, Salem, New Hampshire) were used, respectively. These wavelengths were chosen because they have been shown to favor PSII or PSI in plants (Mattila et al. 2020), and the wavelength specificities of PSII and PSI are mainly determined by the combination of the Chl *a/b* ratios of the photosystems and the absorbance ratio of Chl *a* to Chl *b* at different wavelengths. The Chl *a*/*b* ratio of PSII is lower than that of PSI in both plants (Galka et al. 2012; Wei et al. 2016; Su et al. 2017; Caspy and Nelson 2018; Mattila et al. 2020) and *C. reinhardtii*, although the difference in *C. reinhardtii* is smaller (Drop et al. 2014a, 2014b; Shen et al. 2019).

The specific types of white light were used at low-intensity (PFD 30 µmol m^−2^ s^−1^) to illuminate the cells (grown at PPFD 100 µmol m^−2^ s^−1^) for 5 min, after which the redox state of the PQ-pool was also measured as described above.

In addition to using the two types of white light, the redox state of the PQ-pool was measured after illuminating cells (grown at PPFD 100 µmol m^−2^ s^−1^) for 5 min with the individual wavelength components of the two types of white light (see Fig. S2b for the spectra) at PFD 50 µmol m^−2^ s^−1^.

For the measurement of the PQ-pool redox state in darkness in the presence and absence of oxygen, 10 ml samples with 15 × 10^6^ cells were incubated in the dark for 2 h with continuous bubbling with either air or nitrogen, respectively. Aerobic dark incubations were conducted in 50 ml Erlenmeyer flasks placed on a horizontal shaker. Oxygen concentration was recorded directly from the cell suspension with a FireSting O_2_ Fiberoptic Oxygen Meter (PyroScience GmbH, Aachen, Germany) (see Fig. S3 for the recorded oxygen concentrations). Cell filtering was done by pouring the cells on the filter directly from the flask. Anaerobic incubations were conducted in a sealed chamber to maintain anaerobic conditions in the liquid sample and in the surrounding gas phase. After flushing the chamber with nitrogen, the gas line was submerged in the sample for the duration of the incubation. Anaerobic conditions were confirmed by monitoring the oxygen levels in the gas phase inside the chamber throughout the incubation. Anaerobic incubation was also done in the presence of 5 µM DBMIB that was added to the samples prior to the sealing of the chamber. After the incubations, cells were filtered on a glass microfiber filter while keeping the cells in the dark, sealed chamber and anaerobic atmosphere.

### Fluorescence and P700 measurements

Chl *a* fluorescence and P_700_^+^ absorbance were simultaneously monitored in vivo with Dual-PAM-100 (Heinz Walz GmbH, Effeltrich, Germany). 1.5 ml samples with 40 µg (Chl) ml^−1^ were incubated in the dark for 1 h prior to the experiments. Mixing was provided with a magnetic stirrer. After the dark incubation, the F_V_/F_M_ fluorescence parameter describing the status of PSII, and P_M_, the maximum oxidizable amount of the primary donor of PSI, P_700_, were determined. F_V_/F_M_, defined as (F_M_-F_0_)/F_M_, was obtained by measuring the F_0_ value after dark incubation using only the weak measuring beam of the fluorometer and then firing a 400 ms saturating pulse (PPFD 4000 µmol m^−2^ s^−1^) to measure F_M_. P_M_ was obtained after the F_M_ measurement by illuminating the sample with far-red light for 10 s and then firing a saturating pulse. After determining F_V_/F_M_ and P_M_, 5 min illumination with monochromatic light (PFD 50 µmol m^−2^ s^−1^) was initiated. Saturating pulses were fired at 30 s intervals for the duration of the illumination. From the saturating pulses, yield estimates for PSI (ɸI) and PSII (ɸII) were calculated. ɸI, a measure of the fraction of PSI in which the primary donor, P_700_, was oxidized by the saturating pulse during the illumination with monochromatic light, was calculated as ɸI = (P_M_’–P)/P_M_. ɸII, defined as (F_M_’-F)/F_M_’, was obtained by measuring Chl *a* fluorescence during the illumination with monochromatic light before (F, average of values during the last 0.2 s before the saturating pulse) and during the saturating pulse (F_M_’). Relative electron transfer rates for PSI (rETR(I)) and PSII (rETR(II)) were calculated from the ɸI and ɸII values, respectively, as rETR(I/II) = PFD × ɸ(I/II) × *p* (Miyake et al. 2005), where *p* is the total absorbance value of the sample in the incident light. Equal absorbance of PSI and PSII at all wavelengths was assumed. The *p* value was determined by measuring the absorbance spectrum of the cell suspension with an integrating-sphere spectrophotometer (OLIS CLARiTY 17 UV/VIS/NIR, On Line Instrument Systems, Inc., Athens, Georgia). Absorbance was measured from 8 ml samples with OD_730_ of 0.2 at 23 °C, and absorbance values were calculated according to Fry et al. (2010). The *p* values were then extracted from the measured spectra (Fig. S4) at the wavelengths used, averaged from 4 biological replicates.

### Low-temperature fluorescence emission

Samples for fluorescence spectroscopy at liquid nitrogen temperature were prepared after similar treatments as in the PQ measurements. For the dark incubations, State 1 was induced with air-bubbling and State 2 with anaerobicity, obtained by bubbling the cell suspension with nitrogen. Aliquots of cultures on a horizontal shaker with volume of 10 ml and OD_730_ of 0.5 (18.18 µg Chl ml^−1^) were subjected to the dark conditions for 2 h, after which the final fluorescence samples were taken directly from the treated cell suspensions and diluted to final Chl concentration of 6 µg Chl ml^−1^; preliminary experiments showed no self-absorbance artifact at this Chl concentration. We also tested the combined effect of anaerobic conditions and 5 µM DBMIB, added to the cell suspension prior to the incubation. 0.25 mM sodium fluorescein was added to all samples as an external control prior to freezing in liquid nitrogen, after which the samples were stored at –80 °C until measured. Low-temperature fluorescence emission was measured by exciting the samples with 470 nm blue light at liquid nitrogen temperature. The emission was recorded with a QEPro spectrometer (Ocean Insight, Ostfildern, Germany).

Here, the treatments with the two white wavelength combinations favoring one photosystem were done by first treating the samples taken from growth conditions for 30 min with one type of white light (PFD 30 µmol m^−2^ s^−1^) and then switching to illumination with the opposite white light combination at the same PFD. Samples (OD_730_ of 0.5, equivalent to 18.18 µg Chl ml^−1^), were placed in 2.5 mL cuvettes and mixed with a magnetic stirrer. Aliquots for analyses were collected after 0, 5 and 20 min of illumination with the second white light. Sodium fluorescein (final concentration 0.25 mM) was added to samples diluted to 6 µg Chl ml^−1^ as an internal standard. Fluorescence spectra were recorded at liquid nitrogen temperature as described above.

As it was not possible to design an “opposite” light pre-treatment for monochromatic light, the effects of monochromatic light were compared with the light state measured in the growth conditions. All treatments were done at PFD 50 µmol m^−2^ s^−1^ of monochromatic light. Different growth light intensities were used to see how the change in light quantity affects the light state regulation; the used growth light PPFDs were 100 and 50 µmol m^−2^ s^−1^, as indicated. Control samples were taken directly from growth conditions and diluted to 6 µg Chl ml^−1^. For the illuminations with monochromatic light, cell cultures were diluted to the OD_730_ of 0.5, and 4.5 mL of culture was placed on a small Petri dish (diameter 5.5 cm) to form a ~ 2 mm layer of the cell suspension containing 18.18 µg Chl ml^−1^. Continuous mixing was provided with a magnetic stirrer. Aliquots were collected after 5 and 20 min of illumination and diluted to 6 µg Chl ml^−1^. Sodium fluorescein (final concentration 0.25 mM) was added to samples as an internal standard, and fluorescence spectra were recorded at liquid nitrogen temperature as described above.

### Western blotting

4.5 mL samples with OD_730_ of 0.5, containing 20 × 10^6^ cells, were placed on a small Petri dish (5.5 cm diameter) directly from growth conditions (PPFD 100 µmol m^−2^ s^−1^) and illuminated with monochromatic light (PFD 50 µmol m^−2^ s^−1^) at indicated wavelengths. Samples were collected after 5 or 20 min of illumination. After harvesting, the cells were immediately resuspended in protein extraction buffer containing phosphatase inhibitor (PhosSTOP, Roche Diagnostics, Mannheim, Germany, Cat. No. 04 906 845 001) to prevent changes in phosphorylation. Proteins were extracted from three biological replicates via subsequent freeze–thaw cycles as described by Virtanen et al. (2021). 10 µg of total protein was loaded into wells of a Mini-PROTEAN TGX gel (Bio-Rad Laboratories, Hercules, California, Cat. No. 4561083) and blotted with a specific antibody against a phosphorylated type II LHCII (Agrisera, Vännäs, Sweden, Prod. No. AS13 2705) in 1:40 000 dilution. The secondary antibody, goat-anti rabbit IgG (H + L), alkaline phosphatase conjugate (Life technologies, REF G21079) was used in the final concentration of 1:50 000. Secondary antibody binding was determined via alkaline phosphatase chemiluminescence emission (Perkin Elmer, Boston, Massachusetts, No. NEL602001KT). Developed films were scanned and the bands were quantified with FIJI image processing software (Fiji Is Just ImageJ, v. 1.52) and normalized to the intensity of the respective sample from growth conditions. Equal loading was confirmed by staining the blotted membranes with Coomassie (Bio-Rad Laboratories, Hercules, USA, Cat. No. 1610406).

## Results

### Photochemically active fraction of plastoquinone in C. reinhardtii

**Fig. 1:**
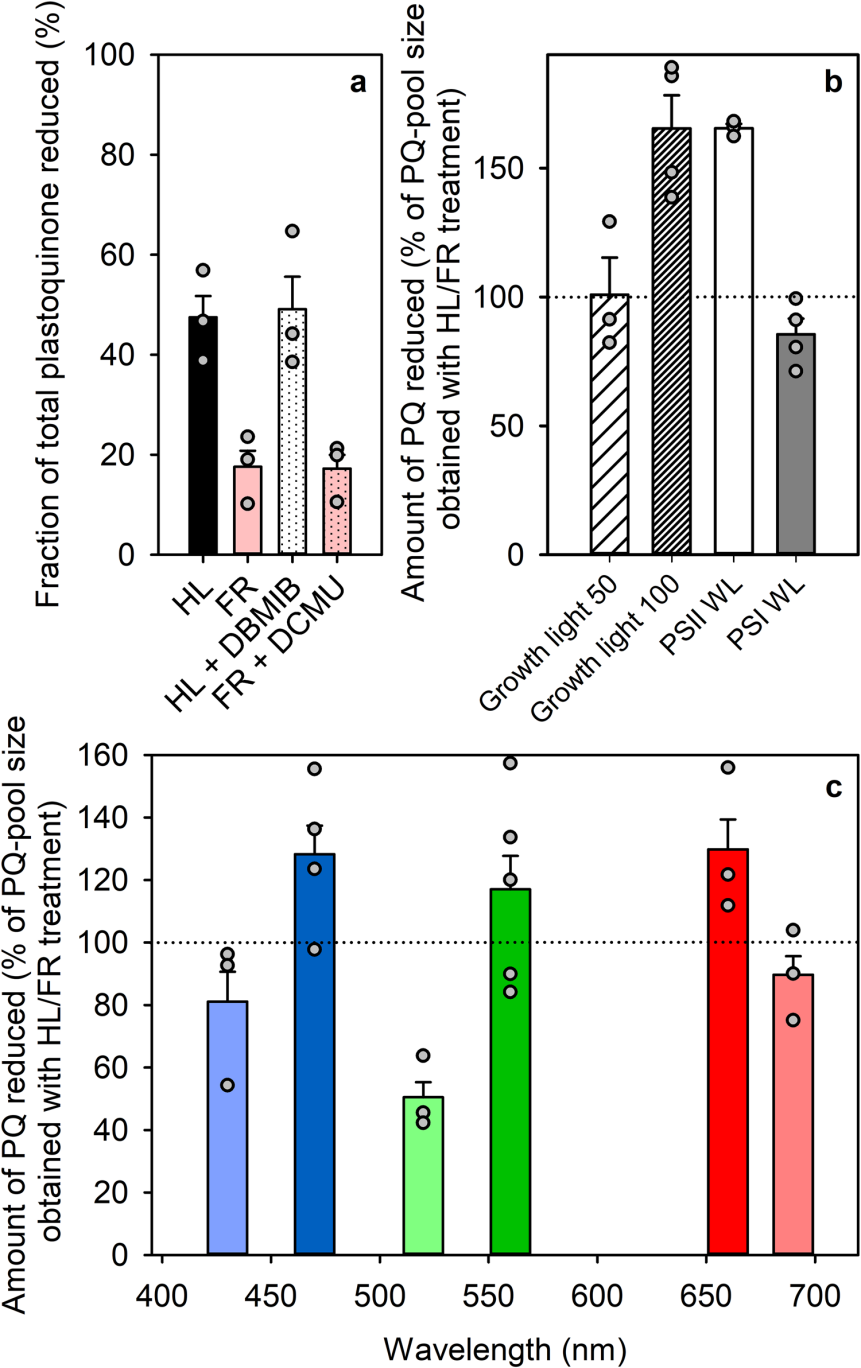
Percentage of reduced PQ in total PQ (**a**), and the redox state of the photochemically active PQ in growth conditions and after treatments with white light favoring PSII or PSI (**b**), and after 5 min under monochromatic light (**c**) in wild-type *C. reinhardtii*. The values in (**b**) and (**c**) show the reduction of PQ according to Eq. (1) in comparison to the size of the PQ-pool, obtained with a high light/FR light treatment shown in (**a**). **a** Approximately 10 – 15 × 10^6^ cells, applied on a filter, were treated for 30 s with strong white light (PFD 2000 µmol m^−2^ s^−1^) (solid, black bar) or 10 min with far-red light (> 700 nm, PFD 50 µmol m^−2^ s^−1^) (solid, light red bar), after which the ratio of photochemically reduced PQH_2_ to total PQH_2_ was measured with HPLC. The measurements were repeated by doing the high light treatment in the presence of DBMIB (dotted, white bar) and the far-red treatment in the presence of DCMU (dotted, light red bar). **b** Cells on a filter were treated for 5 min with growth light at PPFD 50 or 100 µmol m^−2^ s^−1^ (white bars with diagonal stripes). The cells were also grown under these respective light intensities. Similar 5-min treatments were done with white light favoring either PSII (solid white bar) or PSI (solid, grey bar) at PFD 30 µmol m^−2^ s^−1^. The white PSII light was obtained by combining equal PFDs of 470, 560 and 660 nm light, and white PSI light by combining 430, 520 and 690 nm light. **c** Action spectrum of the PQ-pool redox state, measured after 5-min light treatments with monochromatic light at PFD 50 µmol m^−2^ s^−1^, as indicated. Values in b and c were converted from the redox state of the total PQ, according to Kruk and Karpinski (2006), to describe the redox state of the PQ-pool. The dotted line represents the level of 100% reduced PQ-pool, obtained with the short high light treatment. The error bars in all **a**, **b** and **c** show SEM. Each data point represents an average of 3–5 independent biological replicates (circles)

In plants and cyanobacteria, plastoquinone is found in the photochemical PQ-pool of thylakoids, but also in plastoglobuli and the chloroplast envelope membrane. Similar localization is assumed in green algae. We estimated the relative size of the photochemically active fraction with light treatments that successfully reduced or oxidized the PQ-pool in cyanobacteria (Khorobrykh et al. 2020). After a short high light treatment to reduce the PQ-pool, 47.5 ± 5.2% of the total PQ was reduced (Fig. 1a). After maximum oxidation with far-red light (> 700 nm), 17.6 ± 3.9% of total PQ remained reduced. When electron flow through cyt *b*_*6*_*f* was blocked with DBMIB, the PQH_2_ comprised 49.1 ± 7.9% of total PQ after the high light treatment, and when electron flow from PSII was blocked with DCMU and far-red light was used to oxidize the PQ-pool, 17.2 ± 3.4% of total PQ remained reduced. The similarity of the two sets of values shows that the method works; we chose the PQ-pool size obtained without the chemicals, 29.9 ± 9.2% of total PQ, as the basis for further calculations. With this estimation, the PQ-pool in the thylakoids was determined to be fully reduced if at least 47.5% of the total PQ in the cells was reduced, and the PQ-pool was considered completely oxidized if reduction of total PQ was 17.6%.

### In the light, a large fraction of the PQ-pool remains reduced, regardless of light quality

Under growth conditions, light was supplied with a white LED (Fig. S1) containing the whole spectrum of visible light. At PPFD of 50 µmol m^−2^ s^−1^, 100.9 ± 14.4% of the PQ-pool (or 50% of the total PQ) was reduced. Interestingly, even though the cells were grown under the respective light intensities prior to the sample preparation, the increase in the light intensity to PPFD 100 µmol m^−2^ s^−1^ also increased the fraction of reduced PQ to 165.5 ± 12.8% of the size of the PQ-pool obtained with the high light /FR light method, i.e., total PQ contained more PQH_2_ than found after full reduction of the PQ-pool.

When using light that is preferentially absorbed by one of the photosystems, the inter-photosystem electron carriers become reduced or oxidized to a degree that depends on the balance of rates of electron flow through PSII and PSI, respectively. After 5-min illumination with white PSII-favoring light, a fraction of PQ corresponding to 165.5 ± 1.7% of the size of the photochemically active PQ-pool, as measured earlier comparing a short treatment with high light and treatment with FR light, was reduced (Fig. 1b). Conversely, 85.5 ± 6.1% of the PQ-pool, measured with the high light / FR light method, was calculated to have remained reduced after illumination with white PSI light.

We also measured the redox state of the PQ-pool after 5-min illumination with six different wavelengths of monochromatic light, previously shown to favor either PSI or PSII in plants (Mattila et al. 2020). After treatments in green light wavelengths favoring PSII or PSI (560 nm and 520 nm, respectively), 117.0 ± 13.7% and 50.5 ± 6.7% of the apparent size of the photochemically active PQ-pool was reduced, respectively (Fig. 1c). Blue and red PSII/PSI wavelength pairs yielded smaller differences than the green pair: 430 nm blue light favoring PSI reduced 81.1 ± 13.4% and PSII-favoring 470 nm 128.3 ± 10.8% of the amount of photochemically active PQ, as determined above. 129.8 ± 13.4% and 89.7 ± 8.3% of the thylakoid PQ was reduced in 660 nm PSII light and 690 nm PSI light, respectively. The chlorophyll fluorescence parameter qL (Kramer et al. 2004) stayed at 0.9–1.0 during the monochromatic light treatments, indicating that PSII reaction centers remained essentially open (Fig. S5).

### Relative electron transfer rates depend on light quality

**Fig. 2:**
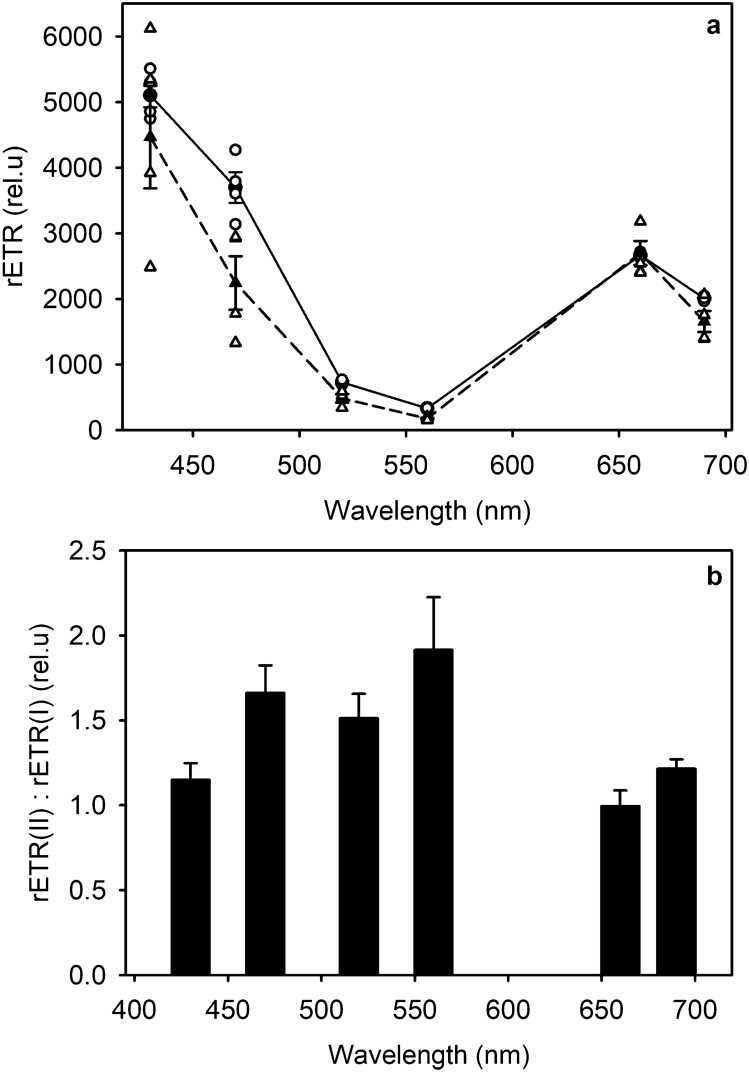
Chlorophyll *a* fluorescence-based relative electron transfer rates through PSI (triangles, dashed line) and PSII (circles, solid line) in *C. reinhardtii* in monochromatic light (**a**) and the ratios between these two relative electron transfer rates at their respective wavelengths (**b**). The relative electron transfer rates were measured during 5-min illumination with monochromatic light (PFD 50 µmol m^−2^ s^−1^). 1.5 ml samples with 40 µg ml^−1^ chlorophyll were incubated in darkness for 1 h, after which they were treated with monochromatic light for five minutes, during which a saturating pulse (PFD 4000 µmol m^−2^ s^−1^, 400 ms) was fired every 30 s from starting the illumination. In a, solid symbols represent an average from ɸ(I/II) x PFD x *p* averaged values measured during the duration of the whole light treatment and open symbols show the values of 3–4 individual biological replicates. The error bars show SEM

Different wavelengths drive photosynthetic reactions at different rates due to wavelength-specific absorption properties of the photosynthetic machinery (Hershey 1995). Inspection of the relative electron transfer rates showed that the difference in the rETR(I) and rETR(II) varied between the wavelengths used here (Fig. 2a). After correcting the values with total absorbance, green wavelengths were the least and the blue ones the most efficient. The rETR(II)-to-rETR(I) ratios show that blue and green wavelengths that caused strong reduction of the photochemically active PQ-pool (470 and 560 nm) also induced a notably higher rETR(II)-to-rETR(I) ratios (Fig. 2b), 1.66 and 1.91, respectively. In turn, both of the PSI wavelengths of respective colors (430 and 520 nm) that caused less PQ-pool reduction, also induced a lower ratio. Interestingly, even though the difference in PQ-pool reduction between the two red wavelengths (660 vs 690 nm) was similar as in green and blue wavelengths, the rETR(II)-to-rETR(I) ratio was not significantly different between 660 and 690 nm, under both of which the ratio resembled that obtained with the blue and green PSI wavelengths (Fig. 2b). The rETR(II) values should be considered as descriptive data rather than exact values, as the recent findings about variable fluorescence (Sipka et al. 2021) and about the unresolved quenching mechanisms affecting the measurements of rETR in microalgae (Havurinne et al. 2019) show that their theoretical basis requires revision.

### Darkness induces strictly Stt7-dependent state transitions that depend on the reduction of the PQ-pool

**Fig. 3:**
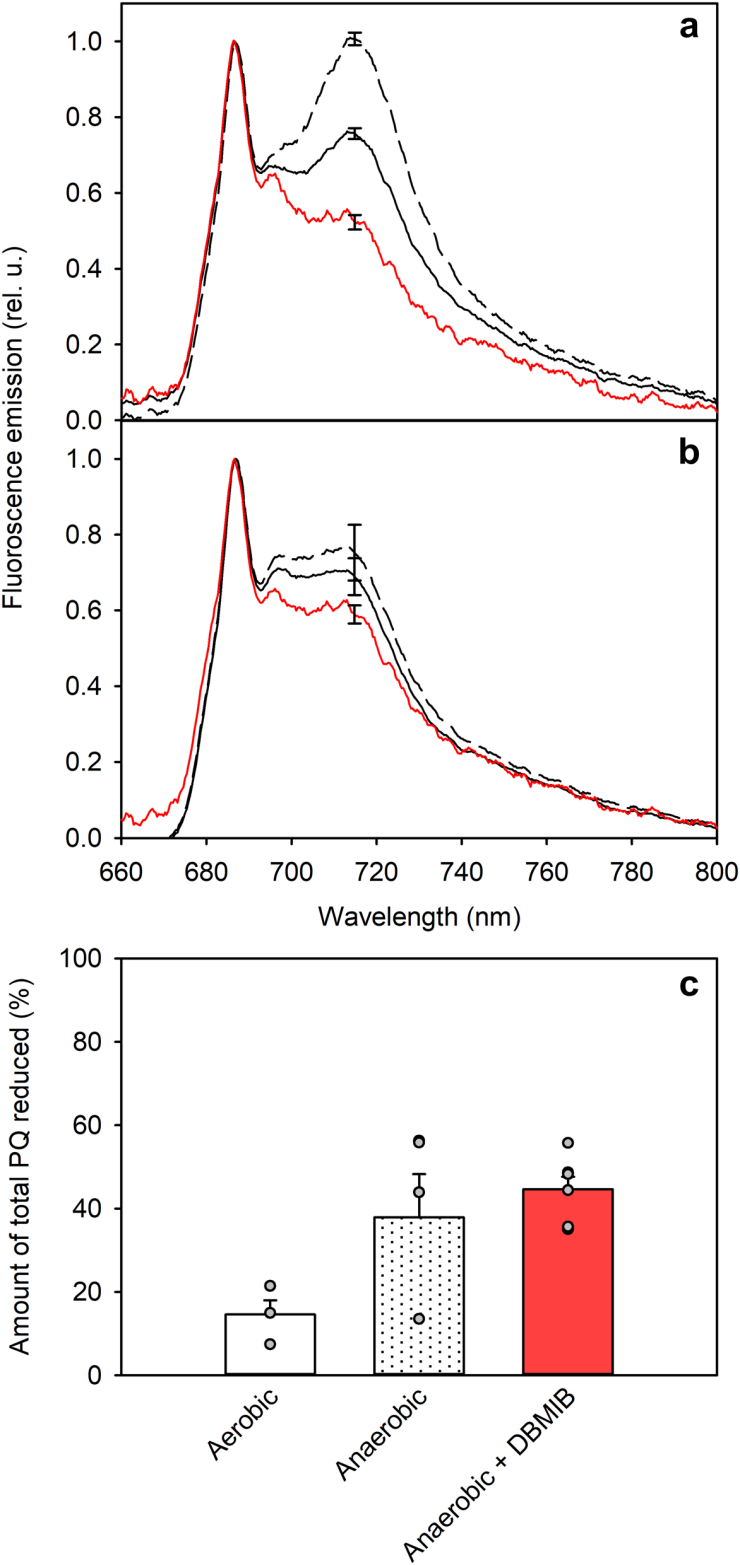
Low-temperature fluorescence emission of wild-type (**a**) and *stt7-9* (**b**) *C. reinhardtii* cells, incubated for two hours aerobically in darkness to induce State 1 (solid, black line), anaerobically in darkness to induce State 2 (dashed, black line) or anaerobically in darkness in the presence of DBMIB (solid, red line); the percentage of reduction of the PQ-pool in the wild-type after similar dark incubations (**c**). Cells were diluted to OD_730_ of 0.5 in a final volume of 10 ml, after which they were placed in the darkness and gas line was submerged in the cell suspension. After treatment, samples for low-temperature fluorescence emission were immediately diluted to the Chl concentration of 6 µg ml^−1^, had 0.25 mM sodium fluorescein added and frozen in liquid nitrogen. Fluorescence emission induced with 470 nm excitation was measured at liquid nitrogen temperature. Samples for PQ measurements were rapidly filtered on a filter in the dark while maintaining the ambient oxygen level of the treatment, after which PQ was extracted and measured with HPLC. Spectra in a and b are normalized to the values at 686 nm and averaged from 4 biological replicates. The error bars at 714 nm show SD. In a and b, statistically significant differences in the F_686_/F_714_ ratio between the traces, examined with Student’s t-test, are shown in the insets (*P < 0.05, **P < 0.005, ***P < 0.0005). Values in c have been averaged from 3–4 biological replicates, values of which are shown as gray circles, and the error bars show SEM

State transitions in *C. reinhardtii* are traditionally induced via dark incubation either in anaerobic conditions to induce State 2 (Delphin et al. 1995; Gans and Wollman 1995; Finazzi et al. 2001, 2002; Forti and Caldiroli 2005; Kargul et al. 2005; Lemeille et al. 2009; Ünlü et al. 2014; Cariti et al. 2020) or by aerating the sample to induce State 1 (Finazzi et al. 2001, 2002; Forti and Caldiroli 2005; Kargul et al. 2005; Ünlü et al. 2014; Cariti et al. 2020). These conditions change the ATP-demand of the cell (Bulté et al. 1990) and alter the NADPH concentrations and chlororespiration rates (Gans and Rebeille 1990; Endo and Asada 1996; Jans et al. 2008). Anaerobic conditions cause the cells to switch to catabolic metabolism and lead to cessation of chlororespiration and increase in PQ-reducing equivalents originating from fermentation. However, to our knowledge, the PQ-pool redox state has not been directly measured after these treatments. Hence, we compared PQ-pool redox state and fluorescence emission spectra after anaerobic and aerobic dark treatments. In aerobic darkness, the wild-type cells showed characteristics of State 1, the default state for the LHCII-phosphorylation deficient mutant *stt7-9* (Fig. 3a, b), and only 14.6 ± 4.0% of total PQ remained reduced (Fig. 3c). After incubation in anaerobic conditions in the dark, the reduction of total PQ had increased to 42.4 ± 10.0%, indicating that 82.8 ± 33.6% of the PQ-pool was reduced. These incubations also induced state transitions in the wild-type, seen as a decrease in the PSII-to-PSI fluorescence emission ratio from 1.32 ± 0.03 observed in aerobic darkness to 0.99 ± 0.02 in anaerobic darkness. The fluorescence ratio is obtained by dividing the 686 nm emission of PSII core (Ferroni et al. 2011) by the 714 nm emission of PSI core (Garnier et al. 1986). The same treatment caused no significant changes in the fluorescence ratio in *stt7-9*. All *P* -values are reported in Table S1. Anaerobic dark incubations were repeated in the presence of DBMIB to confirm that the PQ-pool did not get oxidized during the extraction. Addition of DBMIB increased the reduction of total PQ in anaerobic conditions closer to the maximum measured with the short high light treatment, 44.6 ± 3.3% of total PQ reduced, corresponding to 90.4 ± 11.0% reduction of the PQ-pool (Fig. 3c). However, in the presence of DBMIB the PSII-to-PSI fluorescence ratio increased in the wild-type to 1.90 ± 0.07, and in *stt7-9* to 1.68 ± 0.07, values higher than those measured in State 1 observed after aerobic dark incubation in both the wild-type and *stt7-9*.

### Moderate white light favoring one photosystem induces Stt7-dependent state transitions

**Fig. 4:**
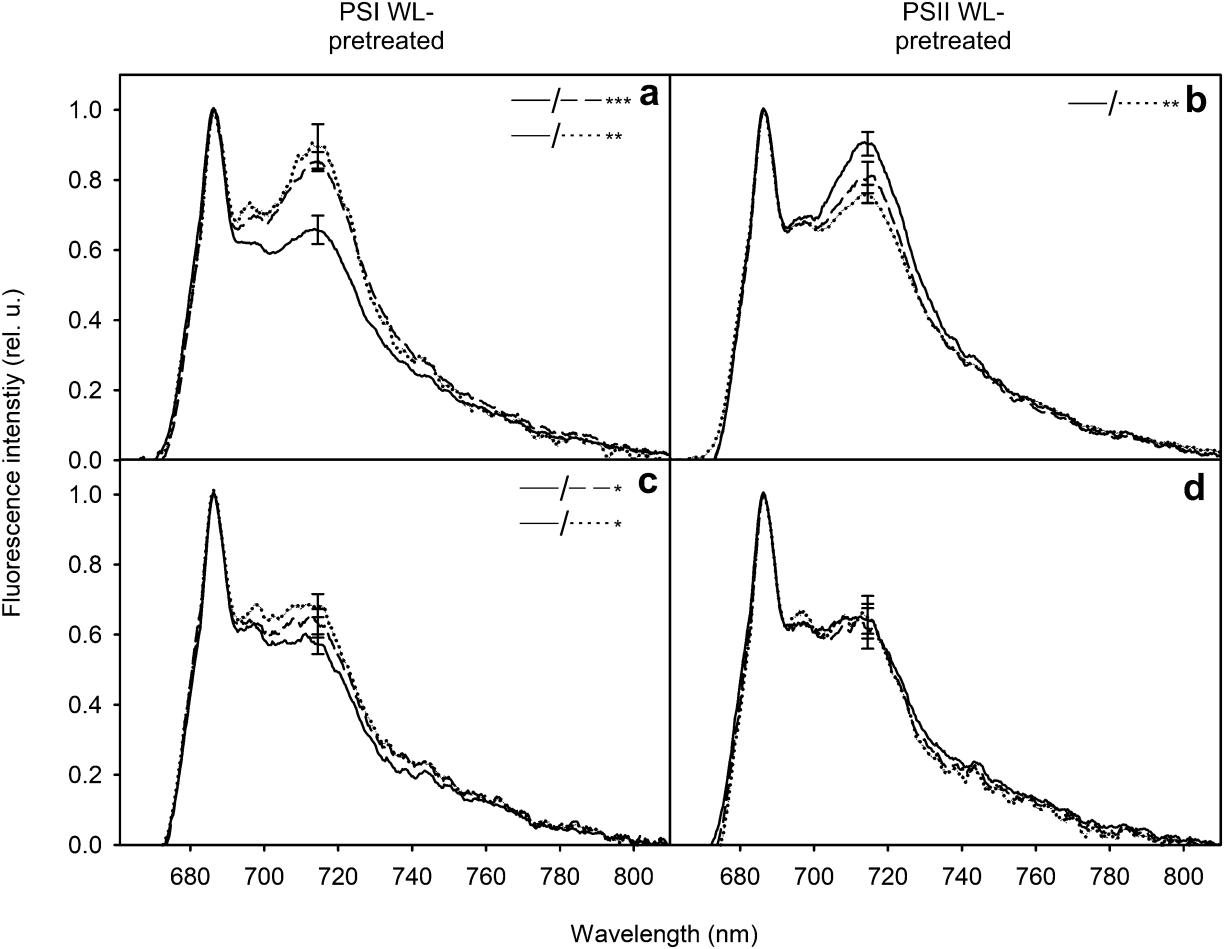
Low-temperature emission spectrum of the wild-type (**a, b**) and *stt7-9* (**c, d**) *C. reinhardtii* cells treated with low-intensity white light favoring the excitation of either PSII (**a, c**) or PSI (**b, d**) after 0 (solid lines), 5 (dashed lines) and 20 (dotted lines) min of illumination. Samples with OD_730_ of 0.5 and chlorophyll concentration of 18.18 µg ml^−1^ were put in a cuvette containing a magnetic stirrer for mixing, and pre-treated first for 30 min with white PSII or PSI light at PFD 30 µmol m^−2^ s^−1^, after which they were transferred to the indicated, opposite white light for 20 min. Aliquots for low-temperature fluorescence spectroscopy were collected after 0, 5 and 20 min in the latter white light. The samples were immediately diluted to the chlorophyll concentration of 6 µg ml^−1^, had 0.25 mM sodium fluorescein added to them and frozen in liquid nitrogen. Fluorescence emission at 470 nm excitation was measured at liquid nitrogen temperature. Each curve represents an average of 3–4 independent biological replications and the error bars show SD. Statistically significant differences between the data at 714 nm, examined with Student’s t-test, are shown in the insets (*P < 0.05, **P < 0.005, ***P < 0.0005)

In addition to the dark incubations, we examined changes in the light state during illumination with white light that favors PSII and strongly reduces the PQ-pool, or favors PSI, causing less reduction of the PQ-pool (Fig. 1b). Illumination with white PSII or PSI light resulted in almost similar changes in the low-temperature fluorescence emission spectrum as anaerobic or aerobic dark incubation, respectively (Figs. 4 and 3a, b). 20-min illumination with white PSII light (after 30-min preillumination with white PSI light) changed the PSII-to-PSI fluorescence ratio (F_686_/F_714_ ratio of low-temperature fluorescence emission) from 1.52 ± 0.08 to 1.12 ± 0.08 (a decrease of 26.5 ± 3.7%) in the wild-type (Fig. 4a) and from 1.76 ± 0.09 to 1.47 ± 0.09 (a decrease of 16.4 ± 6.1%) in *stt7-9* (Fig. 4c). In contrast, treatment with white PSI light after 30-min preillumination with PSII light caused an increase in the PSII-to-PSI fluorescence ratio from 1.11 ± 0.04 to 1.32 ± 0.05 in the wild-type (an increase of 18.9 ± 3.8%) (Fig. 4c), but no observable change in *stt7-9* (Fig. 4d). All *P*-values are reported in Table S2.

### Light quantity over quality: effect of monochromatic lights

**Fig. 5:**
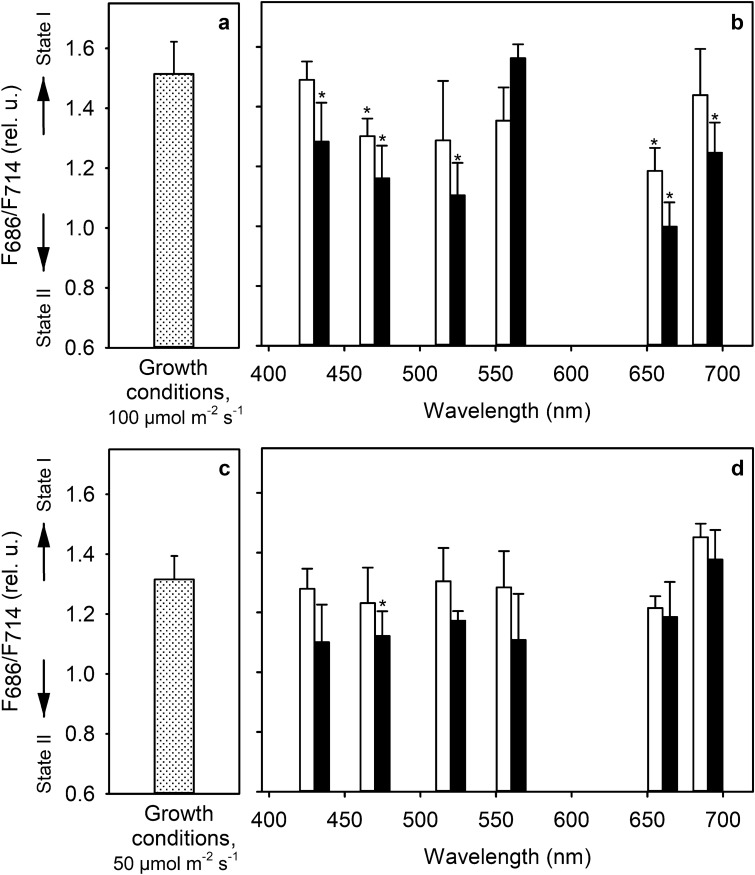
Light state in growth conditions at PPFD 100 µmol m^−2^ s^−1^ (**a**) or 50 µmol m^−2^ s^−1^ (**c**) and effect of monochromatic illumination on light state (**b, d**) in wild-type cells after 5 (white bars) and 20 (black bars) min treatments with monochromatic light of the chosen wavelengths after growth at PPFD 100 (**b**) or 50 µmol m^−2^ s^−1^ (**d**). Samples with OD_730_ of 0.5 and Chl concentration of 18.18 µg ml^−1^ were either taken directly from the growth conditions (**a, c**) or were put on a 5.5 cm Petri dish containing a magnetic stirrer for mixing and were treated for 20 min with monochromatic light at PFD 50 µmol m^−2^ s^−1^ (**b, d**). Aliquots for low-temperature fluorescence emission were collected after 5 and 20 min. Samples were rapidly diluted to the Chl concentration of 6 µg ml^−1^, 0.25 mM sodium fluorescein was added, and frozen in liquid nitrogen. Fluorescence emission was measured at liquid nitrogen temperature via 470 nm excitation. Each bar represents an average of 3–4 biological replicates and the error bars show SD. Asterisks denote statistically significant differences (*P* < 0.05) in F_686_/F_714_ between the growth conditions and after different treatments

We used monochromatic light to test whether wavelengths that caused different levels of reduction of the PQ-pool would also cause similar state transitions in *C. reinhardtii*, as previously observed in *A. thaliana* (Mattila et al. 2020). In the standard growth conditions (PPFD 100 µmol m^−2^ s^−1^), the cells had a PSII-to-PSI fluorescence ratio of 1.51 ± 0.11 (Fig. 5a). Interestingly, nearly all tested wavelengths of monochromatic light (PFD 50 µmol m^−2^ s^−1^), regardless of variation in the PQ-pool reduction (Fig. 1c), turned out to exert a qualitatively similar effect on the light state after 5 or 20 min of illumination (Fig. 5b). The PSII wavelength 470 nm (Fig. 1c) caused the PSII-to-PSI fluorescence ratio to decrease to 1.28 ± 0.13, and 660 nm, another PSII wavelength, caused a decrease to 1.00 ± 0.08 after 20 min of illumination. 5-min illumination caused a smaller decrease. However, 430, 520 and 690 nm illumination, wavelengths favoring PSI, also caused lowering of the PSII-to-PSI fluorescence ratio to 1.28 ± 0.13, 1.10 ± 0.11, and to 1.25 ± 0.10 in 20 min, respectively. 5-min illumination at 430 or 690 nm caused no significant effect whereas 520-nm light caused a similar effect as 470-nm light already after 5 min (Fig. 4b). The only exception was the 560 nm light under which the light state was restored to similar state as in growth conditions after 20 min of illumination regardless of similar PQ-reduction as under the 470 and 660 nm (Fig. 1c).

After growing the cells at PPFD 50 µmol m^−2^ s^−1^, the PSII-to-PSI fluorescence ratio was determined to be 1.32 ± 0.08 (Fig. 5c), 12.80 ± 0.12% lower than observed previously at PPFD 100 µmol m^−2^ s^−1^. Regardless of an already lower fluorescence ratio than in PPFD 100 µmol m^−2^ s^−1^, the overall change in the PSII-to-PSI fluorescence ratio was still shifted towards State 2 after 20 min of illumination. Only 690 nm light caused an increase, albeit not significant, in the F_686_/F_714_ ratio to 1.45 ± 0.06 after 5 min of illumination (Fig. 5d). However, the light state equilibrated during the 20 min of illumination to the same level with the one recorded from growth conditions, 1.37 ± 0.08. Aside from 690 nm light, none of the tested light wavelengths caused any significant changes after the first 5 min of illumination. After 20 min of illumination, all these wavelengths had induced a small transition towards State 2. However, only under 470 nm light, the change after 20 min was statistically significant (*P* < 0.05). All *P*-values are reported in Table S3. Please confirm the section headings are correctly identified.They should be.

### Type II LHCBM phosphorylation in monochromatic light

**Fig. 6:**
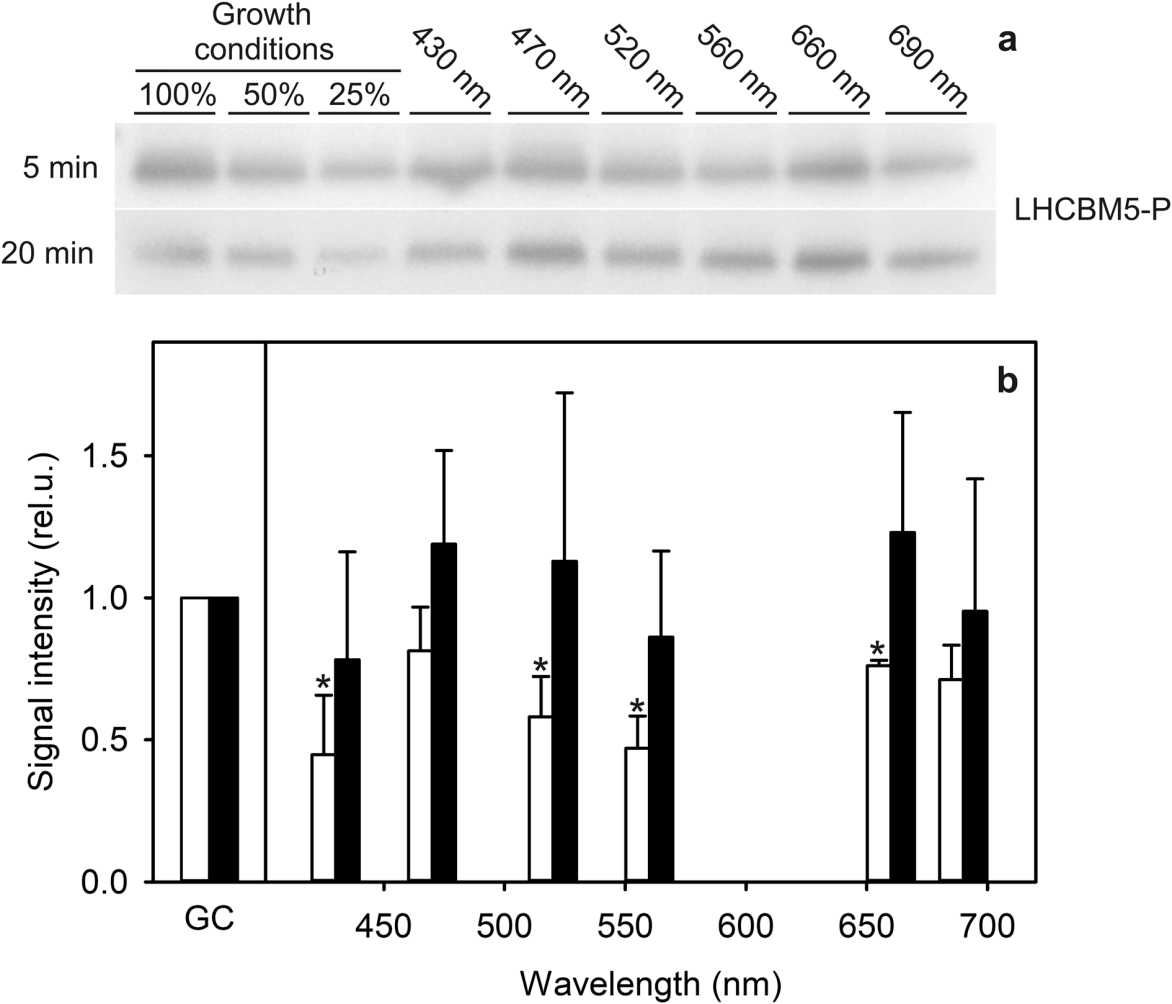
Representative Western blots of phosphorylated type II LHCBM proteins isolated from *C. reinhardtii* samples from growth conditions (GC) (PPFD 100 µmol m^−2^ s^−1^) treated with monochromatic light for 5 or 20 min, as indicated (**a**), and quantification of the bands (**b**). 4.5 ml samples were treated for 5 or 20 min with monochromatic light and total proteins were extracted as described. 10 µg of protein was loaded in each well of SDS-PAGE. Antibody binding was detected with an alkaline phosphatase-based chemiluminescence reaction. The quantified signals were normalized to the control samples, extracted directly from the growth conditions via same method as the treated samples. The bars in b represent average from three biological replicates and the error bars show SD. The asterisks denote statistically significant differences between the samples and the value measured in growth conditions

To further inspect the almost universal response of fluorescence to different wavelengths of monochromatic light, we examined the changes in the abundance of phosphorylated LHCII after the transition from growth light to monochromatic light with a lower PFD. The type II LHCII apoprotein LHCBM5 was chosen because it makes direct contact with PSI core when phosphorylated (Pan et al. 2021) and has been shown to be required for PSI-LHCI-LHCII supercomplex formation in State 2 (Takahashi et al. 2006, 2014). After 5-min illumination, samples from all tested wavelengths showed a lower LHCBM5-P signal than in growth conditions (Fig. 6a). *P*-values for all comparisons are reported in Table S4. However, LHCBM5 phosphorylation was restored in 20-min samples, where the average LHCBM5-P signal was at a similar level as in the growth conditions (Fig. 6b).

## Discussion

### Dynamics of thylakoid-bound plastoquinone in C. reinhardtii

The redox state of the PQ-pool has been recognized as a key regulator of light responses in algae and plants from adjustment of the light state of the photosynthetic machinery to long-term acclimation, including changes in the expression of chloroplastic and nuclear genes (Escoubas 1995; Pfannschmidt et al. 1999; Puthiyaveetil et al. 2008; Ibrahim et al. 2016, 2020). Here we found that the photochemically active PQ-pool in moderate-light grown *C. reinhardtii* cells comprises 29.9 ± 7.5% of total PQ (Fig. 1a), which is slightly less than in plants (Kruk and Karpinski 2006; Yoshida et al. 2010; Mattila et al. 2020) and cyanobacteria (Khorobrykh et al. 2020). In addition, the observation that white PSII light, its monochromatic components and the growth light, cause PQ reduction that highly exceeds the level corresponding to the 100% reduced PQ-pool obtained with a short high light treatment (Fig. 1a) or with a long anaerobic dark incubation with and without DBMIB (Fig. 3c), indicates active exchange of PQ between the photochemical and non-photochemical fractions of PQ during these light treatments. The amount of PQ per cell in *C. reinhardtii* has already been shown to increase in response to extreme light (Virtanen et al. 2021). Hence, the data suggest that *C. reinhardtii* has a mechanism similar to that depending on PGR6 in plants to deploy PQ from plastoglobuli in response to high PQ-reduction (Pralon et al. 2019).

Our data show that *C. reinhardtii* retains a relatively reduced PQ-pool in all illumination conditions applied, aside from far-red light that heavily favors PSI (Fig. 1). Such wavelength dependence suggests that the high reduction state of PQ requires excitation of PSII. In spite of a marked reduction of the PQ-pool, the high value of the qL parameter (Fig. S5) shows that PSII centers need not be photochemically closed. Thus, the fluorescence parameter qL that reflects the redox state of the Q_A_ electron acceptor of PSII (Kramer et al. 2004; Baker 2008), cannot be used to measure the redox state of the PQ-pool in our experiments.

Earlier data suggest that another green alga, *Acetabularia acetabulum*, also maintains the PQ-pool at a rather high reduction state (Havurinne and Tyystjärvi 2020), pointing towards a common trend in green algae. This high reduction state may in part be due to highly active Nda2 in *C. reinhardtii* (Jans et al. 2008; Houyoux et al. 2011). Because Nda2 can utilize reductants in the stroma in a light-independent manner, it may also contribute to the relatively reduced state of the PQ-pool measured after our light treatments lasting for several minutes. Overall, the PQ-pool of *C. reinhardtii* appears to be more reduced in the light than that of *A. thaliana* (Mattila et al. 2020).

In moderate light, the redox state of the PQ-pool is mainly determined by electron transfer rates through PSII and PSI (Mattila et al. 2020), whereas in the dark, reduction by stromal reductants and oxidation via the chlororespiratory pathway dominate. In *A. thaliana*, the visible-light wavelengths used in the present study were found to strongly modulate PQ-pool reduction, whether assayed directly with HPLC or with a fluorescence method (Mattila et al. 2020). In contrast, the fluorescence data here suggest that in *C. reinhardtii*, the same visible-light wavelengths favor electron transfer through PSII over PSI irrespective of the wavelength (Fig. 2). Regardless, differences in the redox state of the PQ-pool can be seen between wavelengths (Fig. 1), indicating that some of the used wavelengths favor PSII less than others.

Photosystem stoichiometry is similar in *A. thaliana* (Wientjes et al. 2017) and in moderate-light grown *C. reinhardtii* (Bonente et al. 2012). Hence, the observed differences in the PQ-pool redox state action spectrum between *C. reinhardtii* and *A. thaliana* (Mattila et al. 2020) are not caused by a different ratio of the photosystems. However, the antenna of PSI has a significantly lower Chl *a*/*b* ratio in *C. reinhardtii* than in plants (van Oort et al. 2008; Galka et al. 2012; Drop et al. 2014a; Casby and Nelson 2018; Suga et al. 2019); this together with the probable broad light absorption of algal photosystems (Tapie et al. 1984; Suga et al. 2019) notably dampens any effect dependent on the differential absorption of Chls *a* and *b* in *C. reinhardtii*. In addition to PSI-LHCI, also LHCII proteins of *C. reinhardtii* have a slightly lower Chl *a*/*b* ratio than those of plants (Drop et al. 2014b). However, the dampened effects of visible-light wavelengths on the redox state of the PQ-pool (Fig. 1c) show that the additional Chl *b* in PSII does not compensate for the notably large contribution of Chl *b* at PSI of *C. reinhardtii*.

In summary, the redox state in *C. reinhardtii* is probably biased towards reduction as a default, probably attributable to more active non-photochemical reduction of PQ in *C. reinhardtii* than in plants. More notably however, the PQ-pool redox states in moderate-intensity lights appear fairly similar at all visible wavelengths because wavelength-dependent differences in the absorption profiles of the two photosystems are small due to a relatively high amount of Chl *b* in the PSI in *C. reinhardtii*.

### Relationship between light state and PQ-pool redox state

The links between chlororespiration (Endo and Asada 1996; Jans et al. 2008), mitochondrial activity (Gans and Rebeille 1990; Cardol et al. 2003) and state transitions in *C. reinhardtii* are well established in the literature. Here we also show how the state transitions and PQ-pool reduction correlate well with each other when induced with dark incubations without chemical additions (Fig. 3). Similar, high levels of reduction of the PQ-pool during the anaerobic dark incubation with and without DBMIB (Fig. 3c) and after short high light treatment (Fig. 1a) suggest that only the photochemically active PQ-pool is affected in the dark, and that the activity of the plastid terminal oxidase, (Houille-Vernes et al. 2011), was negligible during the anaerobic treatments. Furthermore, as no transition to State 2 occurred in *stt7-9* in anaerobic darkness (Fig. 3), the transitions observed in the wild-type can be considered to be dependent on the activity of Stt7. The transition to a deep State 1 in the presence of DBMIB in the dark (Fig. 3a) has been observed earlier (Finazzi et al. 2001) but lacks an explanation and hence we cannot completely rule out possible effects of DBMIB on the fluorescence signal. Regardless, drastic increase in the F_686_/F_714_ ratio in the presence of DBMIB may suggest that DBMIB inhibits Stt7 whereas marginal Stt7-activity is retained even during aerobic dark incubation. Overall, the dark incubations seem to alter only the pre-determined photochemically active PQ-pool, suggesting that the leakage of PQH_2_ from thylakoids, observed under illumination, does not occur in the dark.

As mentioned earlier, green algal state transitions are at least partly contributed to by LHCSRs, especially by LHCSR3. LHCSR3 is activated by a decrease in lumenal pH (Bonente et al. 2011; Tian et al. 2019), occurring as a result of electron transfer in the thylakoids. It is also expected to accumulate in our growth conditions: autotrophy and moderate light (Peers et al. 2009; Tokutsu et al. 2021). Hence, the observed transitions to State 2 in white PSII-light are possibly accompanied by a minor LHCSR3-dependent LHCII detachment from PSII (Roach and Na 2017). When illuminated with white PSII light, an increase is seen in the 694 nm fluorescence corresponding to fluorescence emitted by the PSII-LHCII complex (Ferroni et al. 2011) in both wild-type and *stt7-9*. After 20 min in white PSII light, the 714-to-694 nm fluorescence ratio increases from 1.05 ± 0.05 to 1.25 ± 0.11, *P* < 0.05, in the wild-type (Fig. 4a) and from 0.93 ± 0.04 to 1.04 ± 0.02, *P* < 0.05, in *stt7-9* (Fig. 4c), indicating that the increase at 694 nm may not be entirely due to increase in the wide peak of PSI fluorescence. Hence, with the assumption that *stt7-9* is a slightly leaky mutant (Bergner et al. 2015), after subtracting the argued effect of LHCSR3 under PSII-light, the remaining transitions occurring in the wild-type under the two types of white light depending on Stt7 (PSII-light) and its two antagonistic phosphatases (PSI-light) (Cariti et al. 2020) account for 10–18% of change in the PSII-to-PSI fluorescence ratio and consequently of a similar amount of LHCII moving between photosystems. This result is in line with previous reports (Takahashi et al. 2013; Nagy et al. 2014; Ünlü et al. 2014). In summary, we demonstrate that we can induce almost purely Stt7-dependent state transitions using low-intensity polychromatic visible-light (Fig. 4), while the PQ-pool only varies between a fairly reduced and very highly reduced state (Fig. 1b), a phenomenon not seen in plants (Mattila et al. 2020).

Regardless of differences in PQ-pool reduction (Fig. 1c), almost all monochromatic wavelengths induced a transition towards State 2 from the value measured in the growth conditions (Fig. 5). The restoration of the light state during 20 min under 560 nm light after growth at PPFD 100 µmol m^−2^ s^−1^ (Fig. 5b) is a peculiar observation and lacks clear explanation. Nonetheless, the transitions under all other wavelengths were exaggerated when the light intensity simultaneously decreased during the transition to the monochromatic lights (Fig. 5a, b). The changes in the light state observed after the treatments with white PSII light closely resemble the results after the treatments with its individual components. Conversely, whereas the white PSI light decreased the PSI-related fluorescence peak and caused a transition to State 1, its individual components caused transition to State 2 although the PQ-pool was less reduced in white PSI light and its component wavelengths than in PSII light. In general, the changes under individual wavelengths were seemingly more dependent on changes in light intensity than on the redox state of the PQ-pool, suggesting an additional layer of feedback control of light state. Such control might be important under fluctuating light where LHCSRs and state transitions both have been shown to contribute to NPQ (Steen et al. 2022). Light intensity-dependent feedback in the fluorescence emission data (Fig. 5) was examined via Western blots (Fig. 6). Together these data suggest two phases of response when cells are transferred to lower light intensity than the growth light, more or less regardless of wavelength. First, a rapid lowering in light intensity leads to a cessation of net phosphorylation of LHCBM5. The changes during this phase are probably caused mainly by LHCSR3 that rapidly modulates the functional size of the PSII antenna (Roach and Na 2017). The LHCBM5-P abundance is restored during the second phase, ending after 20 min.

The observations discussed above suggest that the light state in *C. reinhardtii* is dependent on two different feedback mechanisms that react to the changes in light intensity and the PQ-pool redox state, respectively. The first mechanism depends on light intensity and probably reflects the previously shown role of algal state transitions in photoprotection (Allorent et al. 2013; Roach and Na 2017), whereas the latter responds to the redox state of the PQ-pool, balancing light utilization between the photosystems in low light.

## Conclusions

The differences between the behavior of the redox state of the PQ-pool in *C. reinhardtii*, compared to those in *A. thaliana*, are most probably due to the larger amount of Chl *b* in PSI in this alga than in plants. The larger proportion of Chl *b* at PSI effectively dampens the differential effects of visible-light wavelengths on the two photosystems. Overall, the PQ-pool in *C. reinhardtii* remains in a highly reduced state in the light, and an active flow of PQH_2_ from thylakoids occurs in the light. In addition, the connection between the redox state of the PQ-pool and light state appears to be more complex in *C. reinhardtii* than in plants, and light quantity, in addition to light quality, plays a major role. Anaerobic and aerobic dark incubation, as traditionally used to control the light state of *C. reinhardtii*, cause similar Stt7-dependent state transitions as white light favoring PSII or PSI, respectively, although the redox state of the PQ-pool responds to the light and dark treatments in a very different way.

## Supplementary Information

Below is the link to the electronic supplementary material.

Supplementary file1 (TIF 10510 KB)

Supplementary file2 (TIF 55808 KB)

Supplementary file3 (TIF 32526 KB)

Supplementary file4 (TIF 32171 KB)

Supplementary file5 (TIF 8824 KB)

Supplementary file6 (TIF 48946 KB)

Supplementary file7 (DOCX 17 KB)

Supplementary file8 (DOCX 16 KB)

## Data Availability

The data reported are available in the Mendeley Data repository (https://doi.org/10.17632/c6m4g9hbr5.1) and from the corresponding author on reasonable request.
