# Supplementary figures and images for: Plastoquinone pool redox state and control of state transitions in Chlamydomonas reinhardtii in darkness and under illumination
Source: Photosynth Res. 2022 Oct 25;155(1):59–76. doi: 10.1007/s11120-022-00970-3 (PMC9792418; doi:10.1007/s11120-022-00970-3)

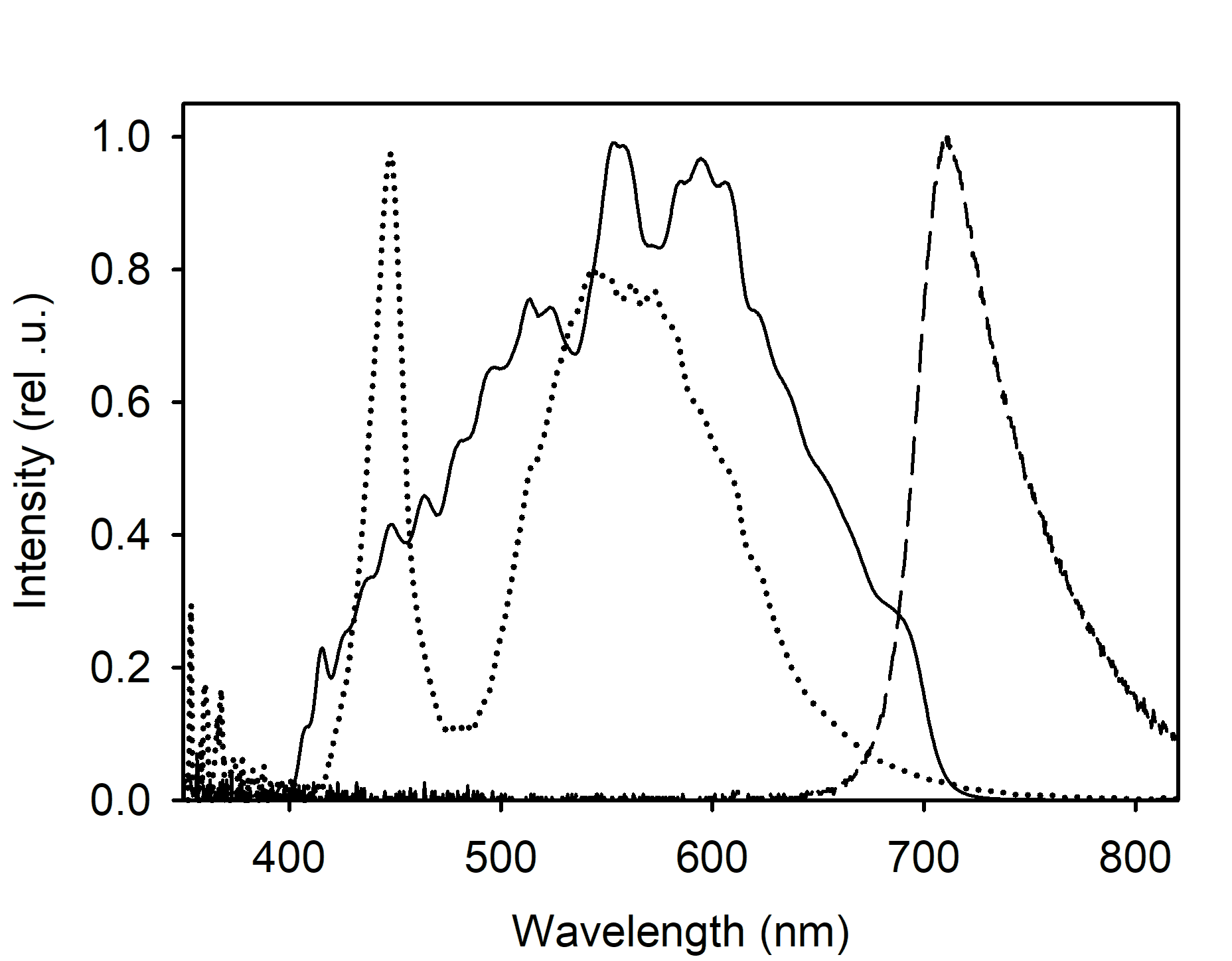

Supplement: Supplementary file 1 — Supplementary file1 (TIF 10510 KB) [file 11120_2022_970_MOESM1_ESM.tif]

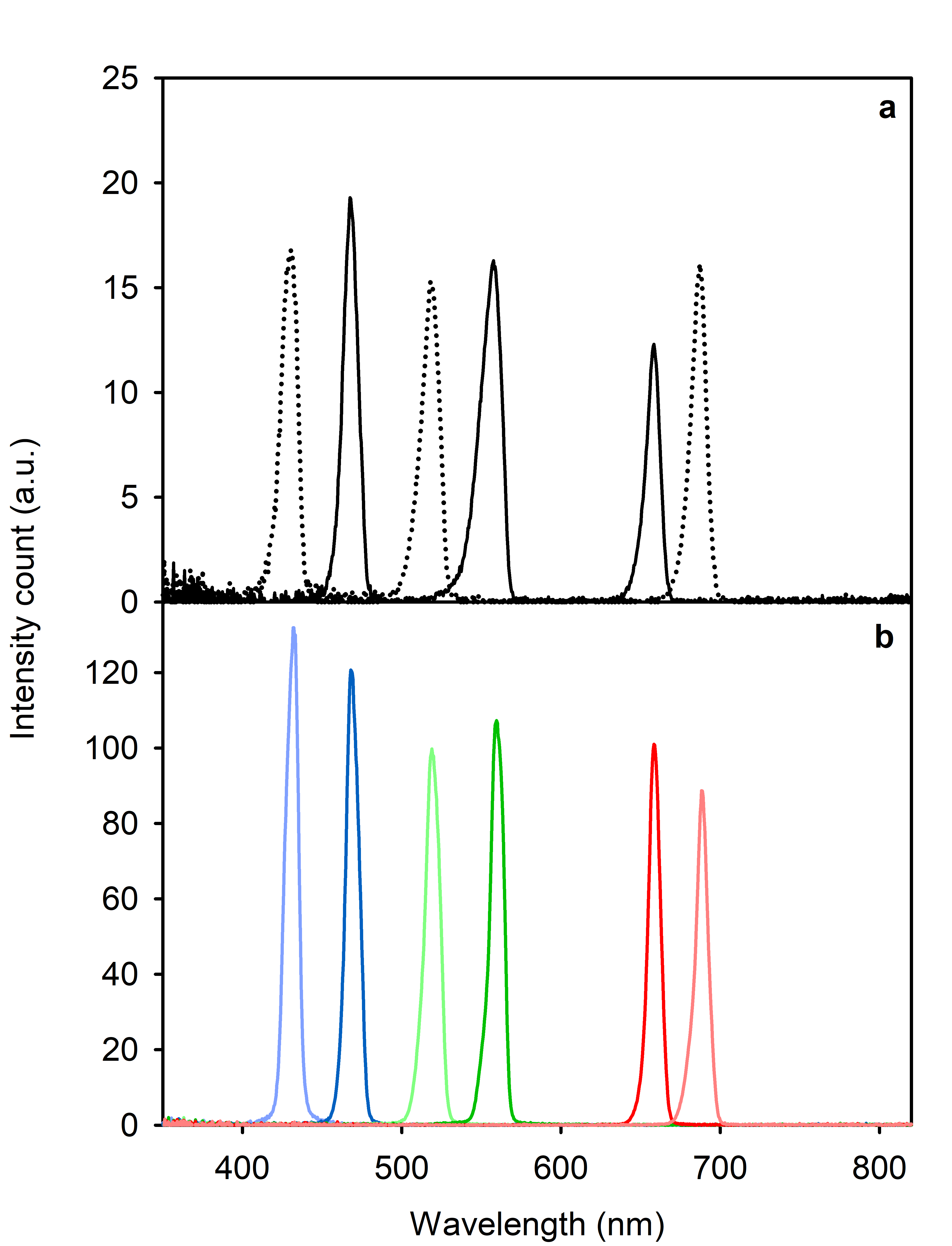

Supplement: Supplementary file 2 — Supplementary file2 (TIF 55808 KB) [file 11120_2022_970_MOESM2_ESM.tif]

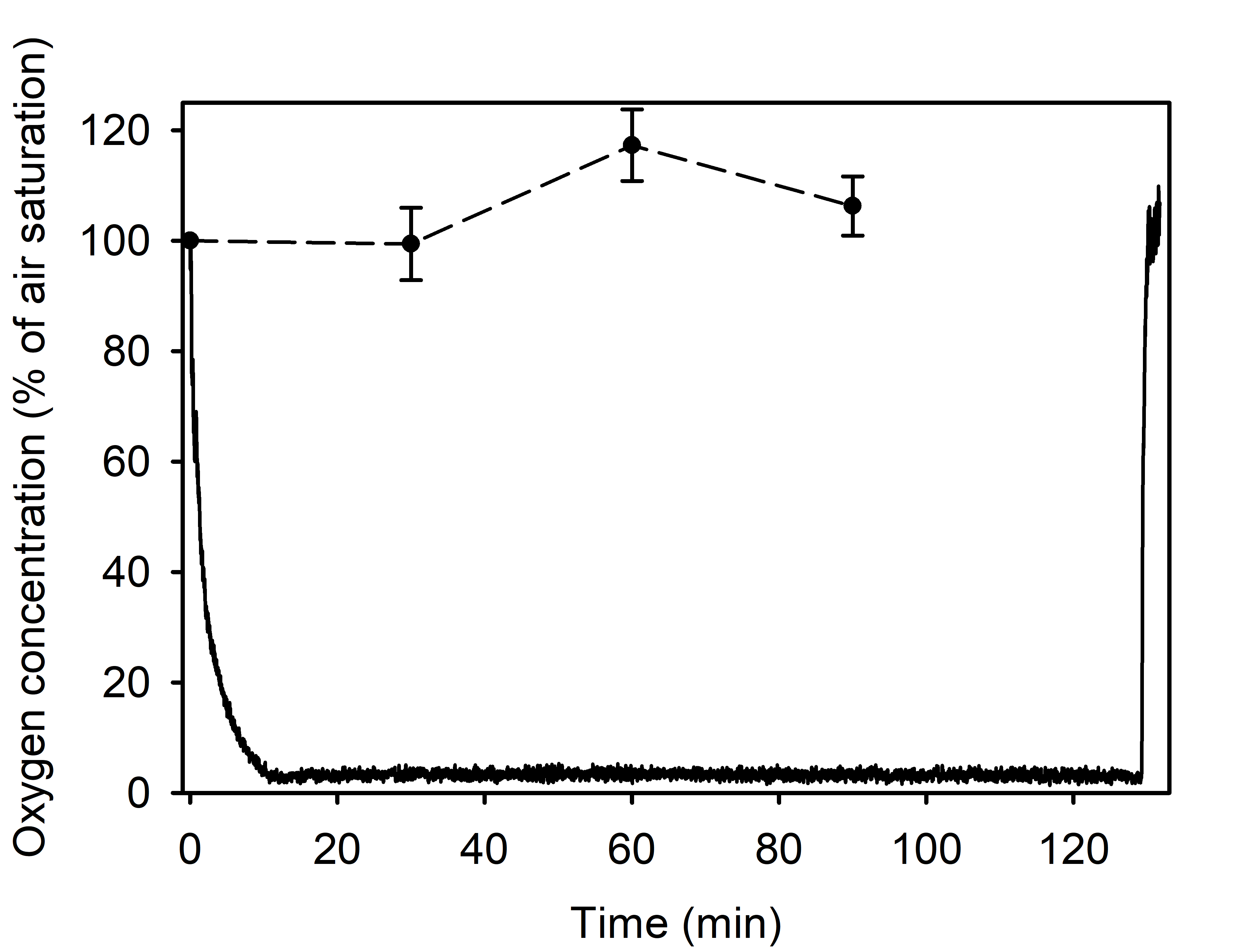

Supplement: Supplementary file 3 — Supplementary file3 (TIF 32526 KB) [file 11120_2022_970_MOESM3_ESM.tif]

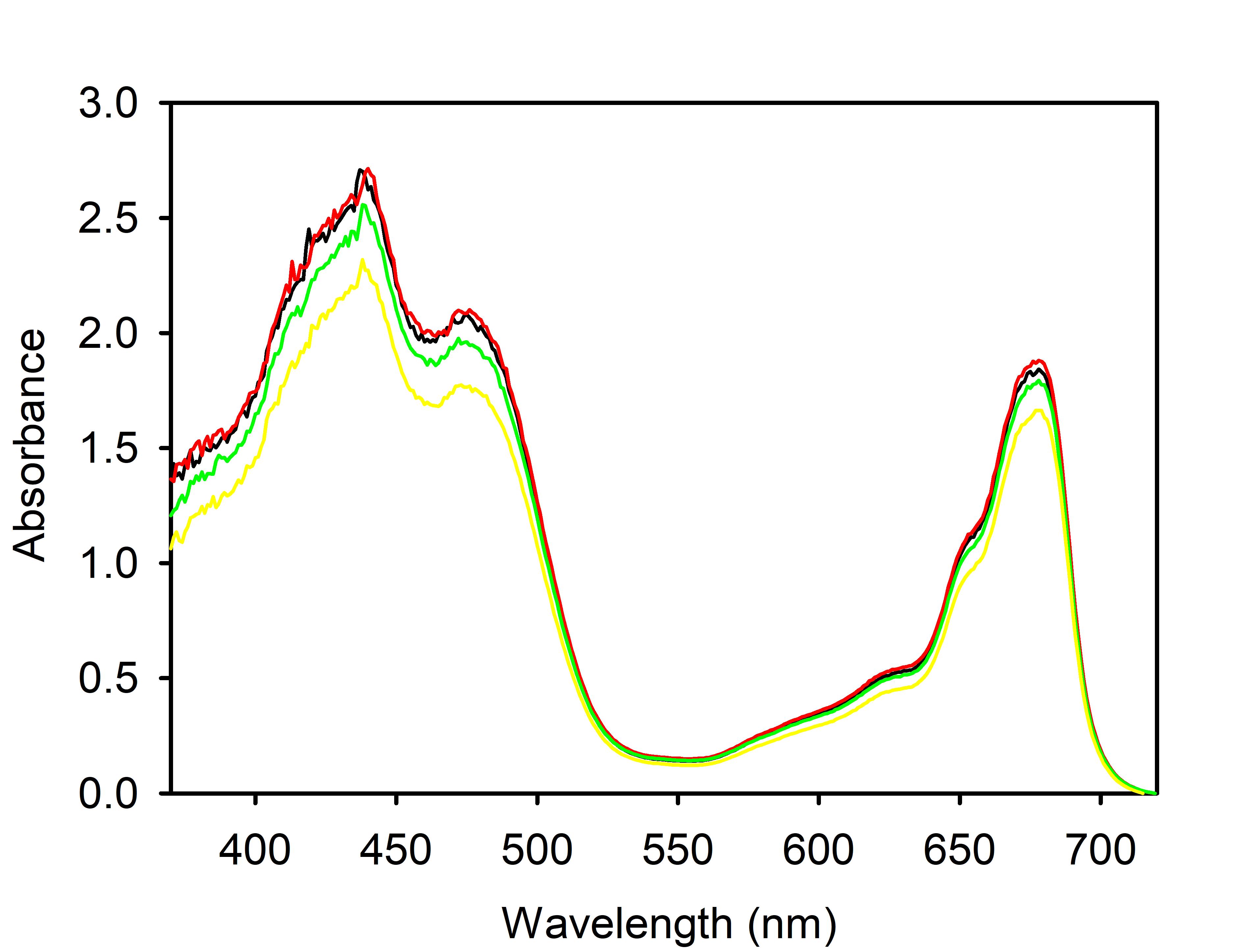

Supplement: Supplementary file 4 — Supplementary file4 (TIF 32171 KB) [file 11120_2022_970_MOESM4_ESM.tif]

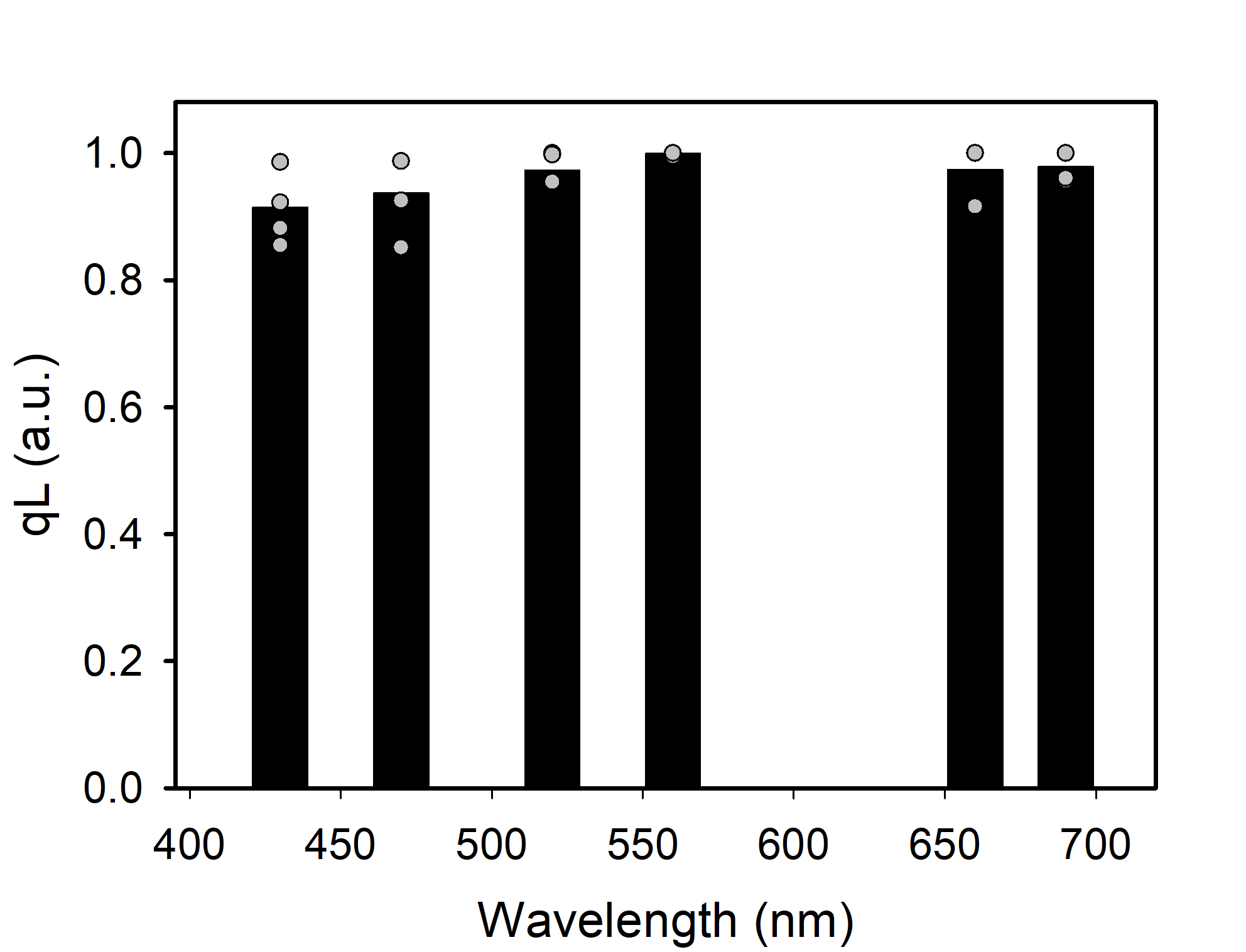

Supplement: Supplementary file 5 — Supplementary file5 (TIF 8824 KB) [file 11120_2022_970_MOESM5_ESM.tif]

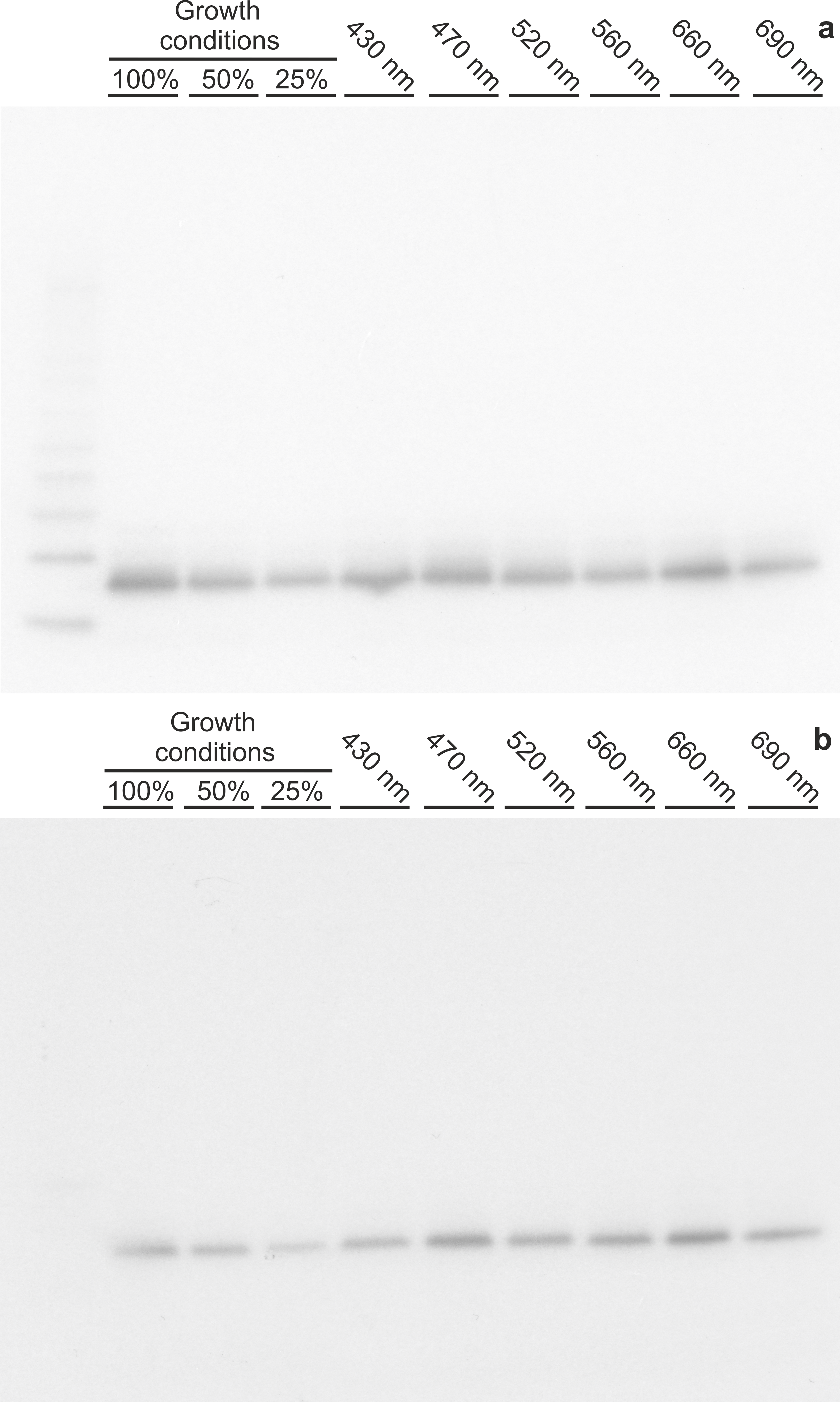

Supplement: Supplementary file 6 — Supplementary file6 (TIF 48946 KB) [file 11120_2022_970_MOESM6_ESM.tif]
