# Supplementary material for: Plastoquinone pool redox state and control of state transitions in Chlamydomonas reinhardtii in darkness and under illumination
Source: Photosynth Res. 2022 Oct 25;155(1):59–76. doi: 10.1007/s11120-022-00970-3 (PMC9792418; doi:10.1007/s11120-022-00970-3)
Supplement: Supplementary file 7 — Supplementary file7 (DOCX 17 KB) [file 11120_2022_970_MOESM7_ESM.docx]

**Plastoquinone pool redox state and control of state transitions in Chlamydomonas reinhardtii in darkness and under illumination**

*Photosynthesis Research*

Olli Virtanen and Esa Tyystjärvi*

*University of Turku, Department of Life Technologies / Molecular Plant Biology, 20014 Turku Finland.*

*Email:* [*esatyy@utu.fi*](mailto:esatyy@utu.fi)

**Table S1** Table reporting the *P*-values of differences in the F_686_/F_714_-ratios in Figure 3, obtained with Student’s t-test.

| **Strain** | **Comparison** | ***P*-value** |
| --- | --- | --- |
| **wild-type (*cc124*)** | Aerobic vs Anaerobic | 0.202 × 10^5^ |
|  | Aerobic vs Anaerobic + DBMIB | 0.120 × 10^3^ |
|  | Anaerobic vs Anaerobic + DBMIB | 0.457 × 10^4^ |
| ***stt7-9*** | Aerobic vs Anaerobic | 0.199 |
|  | Aerobic vs Anaerobic + DBMIB | 0.009 |
|  | Anaerobic vs Anaerobic + DBMIB | 0.005 |

**Table S2** Table reporting the *P*-values of differences in values at 714 nm in Figure 4, obtained with Student’s t-test.

| **Strain** | **Comparison** | ***P*-value** |
| --- | --- | --- |
| **wild-type (*cc124*)** | white PSII light 0 min vs white PSII light 5 min | 0.235 × 10^3^ |
|  | white PSII light 0 min vs white PSII light 20 min | 0.001 |
|  | white PSI light 0 min vs white PSI light 5 min | 0.057 |
|  | white PSI light 0 min vs white PSI light 20 min | 0.531 × 10^3^ |
| ***stt7-9*** | white PSII light 0 min vs white PSII light 5 min | 0.028 |
|  | white PSII light 0 min vs white PSII light 20 min | 0.008 |
|  | white PSI light 0 min vs white PSI light 5 min | 0.479 |
|  | white PSI light 0 min vs white PSI light 20 min | 0.915 |

**Table S3** Table reporting the *P*-values of differences in the F_686_/F_714_ in Figure 5, obtained with Student’s t-test.

| **Growth light intensity** | **Comparison** | ***P*-value** |
| --- | --- | --- |
| **100 µmol m^-2^ s^-1^** | Growth light vs 430 nm 5 min | 0.709 |
|  | Growth light vs 430 nm 20 min | 0.035 |
|  | Growth light vs 470 nm 5 min | 0.020 |
|  | Growth light vs 470 nm 20 min | 0.004 |
|  | Growth light vs 520 nm 5 min | 0.106 |
|  | Growth light vs 520 nm 20 min | 0.002 |
|  | Growth light vs 560 nm 5 min | 0.082 |
|  | Growth light vs 560 nm 20 min | 0.598 |
|  | Growth light vs 660 nm 5 min | 0.003 |
|  | Growth light vs 660 nm 20 min | 0.367 × 10^3^ |
|  | Growth light vs 690 nm 5 min | 0.456 |
|  | Growth light vs 690 nm 20 min | 0.011 |
| **50 µmol m^-2^ s^-1^** | Growth light vs 430 nm 5 min | 0.548 |
|  | Growth light vs 430 nm 20 min | 0.078 |
|  | Growth light vs 470 nm 5 min | 0.307 |
|  | Growth light vs 470 nm 20 min | 0.027 |
|  | Growth light vs 520 nm 5 min | 0.877 |
|  | Growth light vs 520 nm 20 min | 0.070 |
|  | Growth light vs 560 nm 5 min | 0.723 |
|  | Growth light vs 560 nm 20 min | 0.072 |
|  | Growth light vs 660 nm 5 min | 0.140 |
|  | Growth light vs 660 nm 20 min | 0.137 |
|  | Growth light vs 690 nm 5 min | 0.074 |
|  | Growth light vs 690 nm 20 min | 0.452 |

**Table S4** Table reporting the *P*-values for differences to values obtained in growth conditions, shown in Figure 6, obtained with Student’s t-test.

| **Comparison** | ***P*-value** |
| --- | --- |
| Growth conditions vs 430 nm 5 min | 0.045 |
| Growth conditions vs 430 nm 20 min | 0.425 |
| Growth conditions vs 470 nm 5 min | 0.171 |
| Growth conditions vs 470 nm 20 min | 0.425 |
| Growth conditions vs 520 nm 5 min | 0.036 |
| Growth conditions vs 520 nm 20 min | 0.742 |
| Growth conditions vs 560 nm 5 min | 0.015 |
| Growth conditions vs 560 nm 20 min | 0.511 |
| Growth conditions vs 660 nm 5 min | 0.002 |
| Growth conditions vs 660 nm 20 min | 0.444 |
| Growth conditions vs 690 nm 5 min | 0.054 |
| Growth conditions vs 690 nm 20 min | 0.877 |
