# Supplementary material for: Plastoquinone pool redox state and control of state transitions in Chlamydomonas reinhardtii in darkness and under illumination
Source: Photosynth Res. 2022 Oct 25;155(1):59–76. doi: 10.1007/s11120-022-00970-3 (PMC9792418; doi:10.1007/s11120-022-00970-3)
Supplement: Supplementary file 8 — Supplementary file8 (DOCX 16 KB) [file 11120_2022_970_MOESM8_ESM.docx]

**Fig. S1** Spectra of the high light (solid line) and the far-red light (dashed line) used in estimating the photochemically active fraction of the PQ-pool, and the white light in the growth chamber (dotted line), measured with an STS-VIS spectrometer and normalized to the maximum value.

**Fig. S2** (**a**) Spectra of the white light favoring PSII (solid, black line) or PSI (dotted, black line), and (**b**) the monochromatic light sources used to measure the action spectrum of the PQ-pool redox state, each reported in their respective color; the lights were measured and adjusted to PFD of 30 (white PSII or PSI light) or 50 (all types of monochromatic light) µmol m-2 s-1 with an STS-VIS spectrometer.

**Fig. S3** Oxygen concentration during the 2 h anaerobic (solid line) and aerobic (dashed line, closed circles) dark incubation, measured with an optical O_2_ sensor; anaerobic conditions were monitored from the surrounding gas phase and the aerobic conditions directly from the sample.

**Fig. S4** *In vivo* absorption spectra measured with an integrating sphere spectrometer, each line an individual biological replicate similar to that of the ones used in fluorescence measurements, averaged from three technical replicates.

**Fig. S5** qL values calculated from the last saturating flash of the rETR-measurement protocol with Dual-PAM after 5 min of illumination with each wavelength of monochromatic light at PFD 50 µmol m^-2^ s^-1^. The circles show the values of the 3-4 individual biological replicates.

**Fig. S6** Western blots of samples treated for 5 (**a**) or 20 (**b**) minutes with monochromatic light, blotted with LHCBM5-P antibody, binding of which was determined via alkaline phosphatase chemiluminescence emission.
